# Structure Shift of GaN Among Nanowall Network, Nanocolumn, and Compact Film Grown on Si (111) by MBE

**DOI:** 10.1186/s11671-018-2461-1

**Published:** 2018-02-13

**Authors:** Aihua Zhong, Ping Fan, Yuanting Zhong, Dongping Zhang, Fu Li, Jingting Luo, Yizhu Xie, Kazuhiro Hane

**Affiliations:** 1 Shenzhen Key Laboratory of Advanced Thin Films and Applications, College of Physics and Energy, Shenzhen University, Shenzhen, 518060 China; 2 Department of Nanomechanics, Tohoku University, Sendai, 980-8579 Japan; 3 Department of Automotive Engineering, Foshan Polytechnic, Foshan, 528137 China; 4 Shenzhen Key Laboratory of Sensor Technology, Shenzhen, 518060 China

**Keywords:** GaN, Nanowall network, Growth model, Al droplets

## Abstract

Structure shift of GaN nanowall network, nanocolumn, and compact film were successfully obtained on Si (111) by plasma-assisted molecular beam epitaxy (MBE). As is expected, growth of the GaN nanocolumns was observed in N-rich condition on bare Si, and the growth shifted to compact film when the Ga flux was improved. Interestingly, if an aluminum (Al) pre-deposition for 40 s was carried out prior to the GaN growth, GaN grows in the form of the nanowall network. Results show that the pre-deposited Al exits in the form of droplets with typical diameter and height of ~ 80 and ~ 6.7 nm, respectively. A growth model for the nanowall network is proposed and the growth mechanism is discussed. GaN grows in the area without Al droplets while the growth above Al droplets is hindered, resulting in the formation of continuous GaN nanowall network that removes the obstacles of nano-device fabrication.

## Background

As direct wide band gap semiconductors, GaN and related compounds have achieved great success in light-emitting diodes [1–3], laser diodes [4], and high-electron-mobility transistors [5, 6]. The heteroepitaxy of GaN film on sapphire, silicon carbide, or single crystal silicon, however, induces a high density of dislocation. It is believed that their nanostructures have superior performance due to dislocation-free, strain-free, and large surface area-to-volume ratio [7, 8]. Researches on the GaN nanocolumns and nanowires have been intensively carried out [9–12]. The GaN nanocolumnar nucleation occurs spontaneously by Volmer-Weber growth mechanism [13], whereas nitrogen-rich (N-rich) condition prevents the nucleation sites from coalescing. Much attention has been paid to the fabrication of an electrical device on the GaN nanowires or on the nanocolumns. The GaN nanowires were mechanically dispersed on SiO_2_/Si substrate and ohmic contacts formed in two sides of an individual nanowire in random [14]. In another case [15], one side of the nanowire was fixed to a stage connected to negative electrode while another side was aligned to positive electrode by means of scanning electron microscope (SEM), forming an electrical device for scientific research.

Alternatively, a special nanostructure namely the GaN nanowall network which is in-plane electrically conductive is promising since no complex process is needed for the nano-device fabrication. In 2007, growth of the GaN nanowall network was obtained by Kishino’s group using Ti layer patterned by electron-beam lithography as a mask [16]. Several years ago, growth of the GaN nanowall network without any lithography was successfully obtained on sapphire and silicon substrates [17–19]. The band-edge emission intensity of the GaN nanowall network is similar to the GaN nanocolumns and the yellow luminescence is not obvious, indicating high quality of the GaN nanowall network. Different from the separated nanostructure such as the nanocolumns, the nanowall network is in-plane electrically conductive [18, 20, 21] that it could be fabricated into nano-device as easy as the film [22]. Therefore, the obstacle of device fabrication on the separated nanocolumns could be removed by the in-plane electrical conduction of the nanowall network. It is crucial to study the growth mechanism of the nanowall network. Dislocation-induced spontaneous formation of nanowall network is regarded as the growth mechanism of GaN nanowall network on bare *c*-plane sapphire [23]. Since the dislocation-induced formation of nanowall network is of low control, the nanowall network growth on Si (111) substrate with Al buffer layer [18] has been carried out. The growth mechanism of the nanowall on Si (111) is significantly different from that on bare sapphire substrate; however, no research is carried out though the growth mechanism on Si (111) is the key for the nanowall network growth.

In this work, growth of GaN in various structures including the nanowall network, the nanocolumns, and compact film are systematically studied. Various GaN structures mentioned above were grown on Si (111) using plasma-assisted molecular beam epitaxy (MBE). Results show that the structure shift of GaN growth can be achieved by adjusting the Ga/N ratio and by adding the pre-deposited Al droplets. The morphology and photoluminescence of the GaN nanowall network were measured by field emission scanning electron microscopy (FESEM) and photoluminescence spectrum analyzer with He-Cd laser (325 nm, 200 mW) as the excitation source. Atomic force microscope (AFM) was utilized for the characterization of the pre-deposited Al layer. The growth mechanism of GaN nanowall network on Si (111) with metallic Al droplets is proposed.

## Experimental

GaN structures were grown on the Si (111) substrates by Riber 32 MBE system equipped with a N_2_ RF plasma source (Veeco, RFS-N/TH). The pressure of the growth chamber was pumped to 3.0 × 10^− 10^ Torr prior to growth. The N_2_ gas, Ga, and Al with purity of 99.9999% were used in this experiment. Si (111) substrate (without doping, one side polished for growth, 380 ±20 μm, provided by Sigma-Aldrich) with resistivity > 5000 Ω cm was cleaned by standard RCA process, followed by dipping in HF:H_2_O = 1:50 for a few seconds to remove silicon oxide layer on the surface of Si substrate as well as forming a hydrogen-terminated surface.

For the growth of the GaN nanocolumns, the shutters of the N_2_ plasma and the Ga source were simultaneously opened and the bare Si (111) was heated at 973 K. The power and the pressure of the N_2_ plasma source used in all samples in this work were fixed at 400 W and 4.2 × 10^− 5^ Torr, respectively. Prior to the growth of the GaN nanowall network, the Al droplets with diameter of about 80 nm were deposited on the bare Si (111) heated at 973 K. The Al source was kept at 1323 K. The pre-deposition of the Al droplets resulted in a different nucleation and growth of GaN, leading to the growth of the nanowall network. The Ga flux for the growth of the nanowall network was the same with the nanocolumns (*φ*_Ga_ = 1.2 × 10^− 7^ Torr at 1169 K). For the growth of GaN film, the Ga flux was increased to 5.3 × 10^− 7^ Torr while the N flux was kept at constant.

## Results and Discussion

**Fig. 1:**
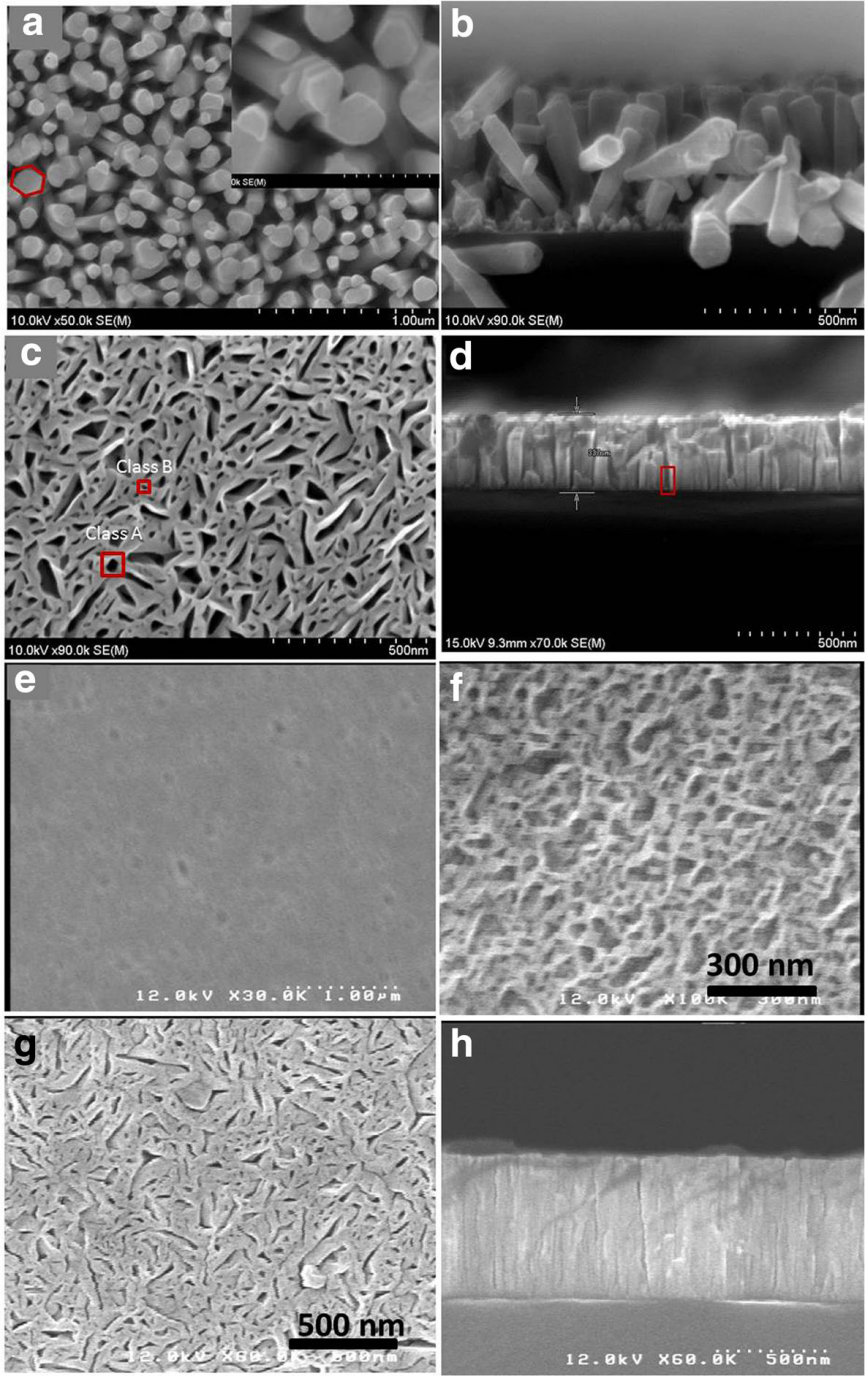
FESEM images of samples grown at different conditions. **a**, **b** Corresponding to the GaN nanocolumns (sample S_1_). **c**, **d** Corresponding to the GaN nanowall network (sample S_2_), **e** Corresponding to the compact film (sample S_3_), **f** Corresponding to the GaN nanowall network (sample S_4_) at the beginning growth stage. **g**, **h** Corresponding to the GaN nanowall network grown at lower temperature 900 K (sample S_5_)

When the shutters of N_2_ plasma and Ga were simultaneously opened, GaN (S_1_) grew in the form of nanocolumns on the bare Si (111) as shown in Fig. 1a. The Ga flux was 1.2 × 10^− 7^ Torr and the Si (111) substrate was kept at 973 K as shown in Table 1. It is observed that the diameter of the GaN nanocolumns ranges from 52 to 125 nm with a length of ~ 460 nm. The density of the nanocolumns counted from SEM image is ~ 10^7^ mm^− 2^. Obviously, most of the nanocolumns observed from Fig. 1b are not perpendicular to the substrate, but tilt with an angle of ~ 30°. The top surface of the nanocolumns is smooth, in consistent with Bertness’s report [9]. It is believed that the nanocolumns nucleate spontaneously and then propagate because the sticking coefficient on the (0 0 01) *c*-plane is higher than that on the {110 0} *m*-plane. The diffusion length *L* of the absorbed Ga atom (Ga_ab_) is essential for the growth of the nanocolumns. As described in Eq. (1), the diffusion length *L* depends on the average jump distance *a*, the Ga_ab_ desorption energy *Q*_des_, and the activation energy for a surface diffusion jump *Q*_d_ [13]*.*

1 $$ L=\sqrt{2}a\ \exp \left(\frac{Q_{\mathrm{d}\mathrm{es}}-{Q}_{\mathrm{d}}}{2 kT}\right) $$

**Table 1:** Summarization of samples studied in this work

Sample	Structure	*φ*_Ga_ (Torr)	Pre-deposition	Growth temperature (K)	Growth time (min)
S_1_	Nanocolumn	1.2 × 10^−7^	Without	973	120
S_2_	Nanowall network	1.2 × 10^−7^	With	973	120
S_3_	Compact film	5.3 × 10^−7^	With	973	120
S_4_	Nanowall network	1.2 × 10^−7^	With	973	20
S_5_	Nanowall network	1.2 × 10^−7^	With	900	120

Since the atomically flat nanocolumn sidewalls provide few adsorption sites, it is assumed that the Ga_ab_ diffusion length *L* on the *m*-plane of sidewalls is much higher than that on the *c*-plane, resulting in the vertical growth of GaN to nanocolumns. If this assumption was true, the strong growth rate anisotropy would be changed when the Ga/N ratio is improved. Indeed, the GaN (S_3_) structure changed from the nanocolumn to the compact film (Fig. 1e) when the Ga flux was increased to 5.3 × 10^− 7^ Torr. Therefore, GaN growth in the form of the nanocolumn or the compact film can be controlled by adjusting the Ш/V ratio.

Though the GaN nanocolumns exhibit superior performance than the film, the fabrication of electrical device is of high difficulty because the separated nanocolumn needs alignment prior to the electrical contact fabrication. An in-plane electrically conductive nanostructure, therefore, is favored. For the growth of sample S_2_, metallic Al pre-deposition was carried out in the MBE growth chamber for 40 s. Then, the N_2_ plasma and the Ga source were simultaneously opened. The Ga flux for the S_2_ growth is summarized in Table 1, the same with that of S_1_. Figure 1c shows the top-view FESEM image of the sample S_2._ It is quite interesting that the GaN grows in the form of the nanowall network on the Al/Si (111). The nanowalls with diameter of 50~100 nm overlap and interlace with one another, forming an in-plane continuous network, namely nanowall network. Two classes of holes are observable, named class A and class B. The diameters of the class A and the class B holes are typically, 50~100 and 10~ 49 nm, respectively. The in-plane continuous characteristic makes the nanowall network in-pane electrically conductive [18], removing the obstacles of nano-device fabrication to some extent. The top surface of the nanowalls is relatively flat, different from the faceted GaN matrix reported in Ref. [13]. It is observable that the holes shown in the top-view image extend to near the substrate, as indicated by the rectangle in Fig. 1d.

One may wonder how the holes mentioned above are generated. We grew a sample S_5_ at a lower growth temperature of 900 K. The other growth parameters are the same with the sample S_2_, as shown in the Table 1. From Fig. 1g, we observe that the sample S_5_ also grew in the form of the nanowall network with smaller holes. Figure 1h is the cross-sectional image of S_5_, showing a more compact layer than S_2._ In order to see the beginning growth of the GaN nanowall network, we grew another sample S_4_ in a short time with Al pre-deposition. All the growth parameters of S_4_ are the same with that of sample S_2_ except for the growth time (20 vs 120 min). The thickness of S_4_ is about 50 nm and its top-view image is shown in Fig. 1f. It is observed that holes have been formed at this stage and the as-gown GaN is an in-plane continuous network, rather than the GaN nanowires or islands. From the study of samples S_1_, S_2_, S_4_, and S_5_, it is clear that the pre-deposition of the Al layer changes the growth behavior of GaN at the beginning, from the nanocolumn to the in-plane continuous nanowall network.

**Fig. 2:**
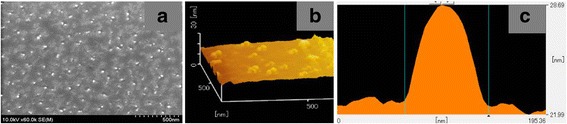
FESEM (**a**) and AFM (**b**) images of the pre-deposited Al on Si substrate. **c** A parameter measurement of one Al droplet by AFM

Note that all growth conditions of S_2_ except Al pre-deposition are the same with that of S_1_. Then, we may wonder what the structure of the pre-deposited Al is and how it affects the subsequent growth of the GaN. To find these answers, Al pre-deposition for 40 s on the bare Si (111) is investigated by FESEM and AFM. Figure 2a shows the top-view image of the pre-deposited Al. It is found that the Al on the Si substrate exists in the form of droplets (white dots) other than film. The metallic Al droplets with density of ~ 4 × 10^7^ mm^− 2^ distribute relative uniformly without significant accumulation. Recently, the Al droplets were successfully grown using MBE by Li et al. to improve the quality of as-grown GaN as well as reducing the stress [24]. To further study the morphology of the Al droplets, AFM was utilized to measure their three-dimensional images and related parameters as shown in Fig. 2b, c. The droplets are sphere as shown in Fig. 2b, in agreement with the SEM result. The height and diameter of the Al droplet measured are 6.7 and 80 nm, respectively. Poppitz et al. [25] have investigated the growth of GaN nanowall network on bare 6H-SiC (0001) by iron-beam-assisted MBE. Their results show that the extremely N-rich growth conditions in combination with the high substrate temperature and the energetic N-ion irradiation during growth are the main reasons for the formation of the nanowall network on bare 6H-SiC (0001). As a pioneer, Kesaria et al. [17] have investigated the GaN nanowall network on bare sapphire substrate using PA-MBE. In their research, it is regarded that the GaN nanowalls nucleate at the edge and the screw dislocations and grow under N-rich atmosphere.

In our case, the growth mechanism of the GaN nanowall network should be different since the nanowalls grow with the requirement of the metallic Al pre-deposition. Of course, to our knowledge, all GaN nanowall networks including in our experiment are grown under the N-rich atmosphere. N rich is required to reduce the coalescence of the nanowalls. Now, let us focus on the role of the Al droplets in the formation of the nanowall network. Similar to the Au droplets acting as a catalyst [26], if the Al droplets acted as a catalyst, the GaN should grow to the nanocolumns rather than the nanowall network. The Al droplets’ role, therefore, is not a catalyst in our study. The melt temperature of the Al metal is 933 K  while the substrate temperature is maintained at 973 K during growth. At the beginning of the GaN growth, thus, the Al droplets are liquid droplets. According to previous report, in the case of GaN on Si (111) with Ga droplets [13], the Ga droplets act as reservoirs that supply Ga atoms to their close vicinity. The Ga droplets themselves, however, hinder the GaN growth on them since the original Ga droplet sites are hollow circles. In our case, the diameter of the Al droplets is ~ 80 nm, similar to the size of the class A holes in Fig. 1a. Therefore, the Al droplets may hinder the growth of GaN on them, leading to the formation of the class A holes observed in the GaN nanowall network. Because of the same Ш/V ratio of samples S_1_ and S_2_, the Ga diffusion length *L* on Si for the nanowall network growth is expected to be the same with that for the nanocolumns. The typical size of the bare Si substrate (the area without Al droplets) is ~ 80 nm, within the value of the nanocolumn diameter in Fig. 1a. In other words, the Ga diffusion length *L* covers the size of the bare Si substrate, resulting in the continuous growth of GaN in the area without the Al droplets, i.e., GaN nanowall network.

**Fig. 3:**
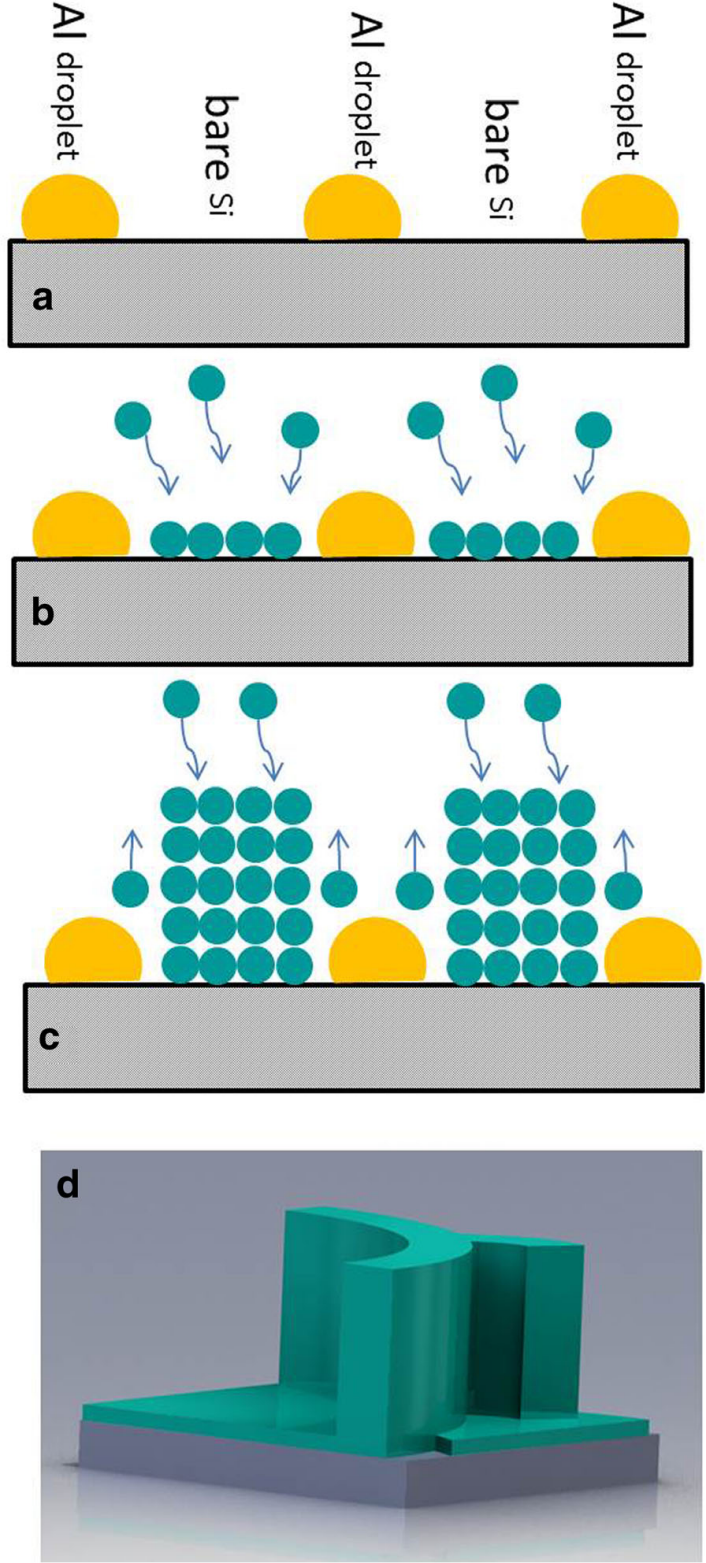
Growth models of the GaN nanowall network. **a** The pre-deposited Al droplets on the Si substrate. **b** Nucleation of the GaN nanowall network on the bare Si. **c** Cross-sectional illustration of the GaN nanowall network grown vertically at the N-rich condition. **d** Tilt illustration of the GaN nanowall network

Basing on the above analyzation, a growth model of the GaN nanowall network is proposed and shown in Fig. 3. GaN nucleates on the bare Si substrate as illustrated in Fig. 3a. Since the Ga_ab_ diffusion length *L* covers the bare Si substrate, GaN grows in the whole bare Si substrate while the GaN growth above the Al droplets is hindered (Fig. 3b). Moreover, under N-rich condition GaN tends to grow vertically as shown in Fig. 3c. Because the bare Si substrate is a continuous network in-plane, the growth of GaN above is also a continuous network named the nanowall network as illustrated in Fig. 3d. This assumption is confirmed by the top-view image of the sample S_4_ in Fig. 1f. Due to the N-rich condition for the sample S_2_ growth, the lateral growth is limited that the holes are able to reserve in the subsequent growth. Note that both the Al droplets and the N-rich condition are essential for the GaN nanowall network growth.

**Fig. 4:**
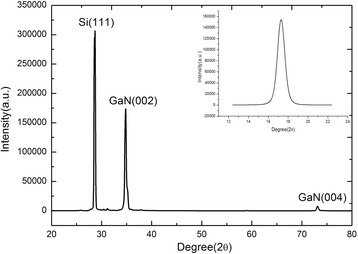
X-ray diffraction pattern of the GaN nanowall network (S_2_). The inset is the ω-scan rocking curve of the sample S_2_

X-ray diffraction was utilized for the crystal structure characterization of the GaN nanowall network as shown in Fig. 4. Two diffraction peaks of the GaN(002) and the GaN(004) are observed together with the Si (111) peak from the Si substrate. The result reveals that the GaN nanowall network is hexagonal, and highly orients along *C* axis. In addition to the XRD pattern, ω-scan rocking curve of the GaN(002) was also measured as shown in the inset of Fig. 4. The full width at half maximum is 52.2 arcmin, similar to the previous report grown on the sapphire substrate [27]. The electrical properties were also measured using Van der Pauw Hall measurement system (HL5500PC, Nanometrics) at 293 K. The electrical conductivity, the hall mobility, and the electron concentration of the GaN nanowall network are 10.2 S/cm, 31 cm^2^/Vs, and 2.1×10^18^ cm^− 3^, respectively. As for the GaN film, the electrical conductivity increases to 666.7 S/cm due to the higher electron concentration of 2.2×10^20^ cm^− 3^. The high carrier concentration in the film is probably attributed to the high intrinsic defect concentration generated by the non-optimized Ga/N ratio. As for the hall mobility of the film, the value is 18.7 cm^2^/Vs.

**Fig. 5:**
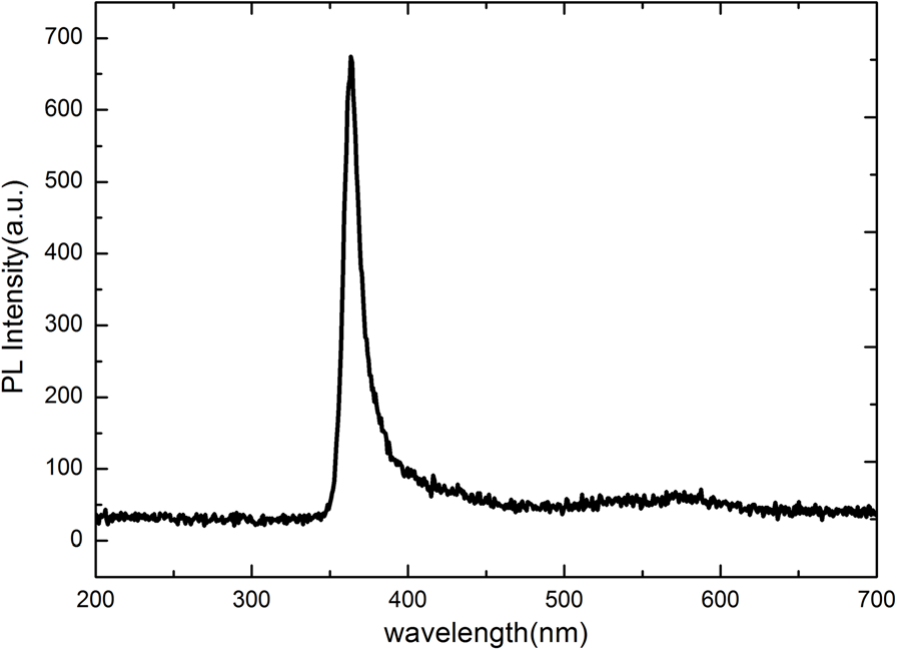
Photoluminescence (PL) spectra of the GaN nanowall network measured at room temperature

Figure 5 shows the photoluminescence spectra of the GaN nanowall network with a He-Cd laser as the excitation source. According to Kesaria et al.’s report [17], direct comparison of cathodoluminescence among GaN film, nanowall network, and nanocolumn grown on bare sapphire substrate was carried out. Their results show that the band-edge emission of nanowall network is slightly higher than that of the nanocolumn, and much higher than that of the film. A broad defect emission centered at 520 to 620 is observable for the nanowall network while no defect emission could be observed for the nanocolumn. In Fig. 5, a strong band-edge emission centered at 363.7 nm is observed with the full width at half maximum of 14.1 nm. In agreement well with Kesaria et al.'s report [17], in the range of 520 to 620 nm, a broad green-yellow emission ascribed to point defects [28] is detectable but very weak, indicating high quality of the GaN nanowall network. Therefore, the luminescence of the GaN nanowall network grown on the bare sapphire substrate and on the Si substrate with the Al droplets are almost the same though the growth mechanism is different.

## Conclusions

In this work, structure shift of the GaN growth among the nanocolumn, the nanowall network, and the compact film was successfully achieved on Si (111) substrate using MBE. The GaN nanocolumns were grown on the bare Si substrate under N-rich condition while the compact film was grown with an improved Ga flux. By adding a pre-deposited Al layer, the growth of GaN shifts from the nanocolumns to the in-plane continuous nanowall network. The pre-deposited Al layer exists in the form of droplets with the typical height and diameter of 6.7 and 80 nm, respectively. The growth mechanism of the nanowall network is addressed. GaN continuously grows on the bare Si substrate while the GaN growth on the Al droplets is hindered, resulting in the formation of the nanowall network. Both Al droplets and N-rich condition are essential for the nanowall network growth.
